# The preventive effect of atorvastatin on liver fibrosis in the bile duct ligation rats via antioxidant activity and down-regulation of Rac1 and NOX1

**DOI:** 10.22038/IJBMS.2019.33663.8047

**Published:** 2020-01

**Authors:** Zohreh-al-sadat Ghoreshi, Razieh Kabirifar, Ameneh Khodarahmi, Alireza Karimollah, Ali Moradi

**Affiliations:** 1 Department of Biochemistry, School of Medicine, Shahid Sadoughi University of Medical Sciences and Health Services, Yazd, Iran; 2 Department of Pharmacology, School of Medicine, Shahid Sadoughi University of Medical Sciences and Health Services, Yazd, Iran

**Keywords:** Atorvastatin, Biliary duct-ligation, Liver fibrosis, NOX1, Oxidative stress, Rac1, Rac1-GTP

## Abstract

**Objective(s):**

Atorvastatin is a cholesterol-lowering agent capable of inhibiting 3-hydroxy-3-methylglutaryl coenzyme A reductase. Recent studies have demonstrated new facets of atorvastatin, such as antioxidant and anti-fibrotic properties. We investigated the effect of atorvastatin on hepatic injury via the measurement of the antioxidant capacity and protein expression of NOX1, Rac1-GTP, and Rac1 in a rat biliary duct ligation (BDL) model.

**Materials and Methods:**

This study is regarded as experimental interventional research in which a total of 32 adult male Wistar rats (200-250 g) were assigned to 4 groups (eight rats per group) as follows: Control group; Control + At group (15 mg\kg\day atorvastatin); BDL group, and BDL+ At group (15 mg\kg\day atorvastatin). Expression levels of Rac1, NOX1, and Rac1-GTP were determined by western blot analysis. Besides, specific biomarkers of oxidative stress in hepatic tissues of all animals were also analyzed.

**Results:**

Atorvastatin reduced liver injury via a decrease in the expression of NOX1, Rac1-GTP, and Rac1 in the BDL group (*P*<0.05), while the increased contents of protein thiol groups were observed, and the protein carbonylation was decreased in atorvastatin-treated BDL rats compared to the BDL group (*P*<0.05). Also, administration of atorvastatin in the BDL group significantly lowered oxidative stress through increasing the activity of catalase and superoxide dismutase in comparison with the BDL group (*P*<0.05).

**Conclusion:**

It seems that atorvastatin has potential advantages in mitigation of liver fibrosis by a decrease in the expression of NOX1, Rac1-GTP, and Rac1, along with, a reduction in oxidative stress of liver tissues in rats induced by BDL.

## Introduction

Bile duct ligation (BDL) is a murine experimental model of cholestasis that results in hepatocellular injuries, fibrogenesis, and eventually, liver fibrosis (1).

During the induction of hepatic fibrosis, desmin-positive hepatic stellate cells (HSC) and other myofibroblastic (MFB) cells become activated and exhibit pro-fibrotic activity. These pro-fibrotic MFB cells generate a high amount of extracellular matrix proteins (e.g., collagen) and pro-fibrotic cytokines such as transforming growth factor-β (TGF-β), connective tissue growth factor (CTGF), and platelet-derived growth factor (PDGF), that promote the progression of fibrosis in an autocrine and paracrine manner (1, 2). 

Clinical and experimental studies demonstrated that oxidative stress critically contributes to the exacerbation of fibrosis, and it can serve as a mediator playing a critical role in liver fibrosis. Generation of reactive oxygen species (ROS) plays an essential role in the activation of HSC and the onset of hepatic fibrogenesis (3). Over the last decade, ROS have also been recognized as critical signaling molecules, modulating the transcription of numerous genes via the activation of redox-sensitive protein kinases and transcription factors. While ROS are produced in enzymatic and non-enzymatic reactions, NADPH oxidase was recognized as a significant source of ROS generated in response to different stimuli. NADPH oxidase is a multicomponent enzyme that consisted of the membrane-bound catalytic subunits, namely NOX and p22phox, which are functionally connected with several cytosolic regulatory subunits (4). HSCs and hepatocytes express NOX1, NOX2, and NOX4 (5). HSCs lacking the NOX1 gene generate fewer ROS compared to HSCs, which are knockout for NOX2; so, the role of NOX1 in the generation of ROS in HSCs is seemingly more pronounced when compared to NOX2 (6).

Rac1 belongs to a sub-family of small GTP-binding proteins including Ras-related C3 botulinum toxin substrate 1 (Rac1), Rac2, Rac3, and other Rac-related proteins. The Rac1 gene regulates many cellular processes such as cell-cell adhesion, cell cycle, and motility (7) and has multiple downstream mediators such as NOX and various cell surface receptor-mediated signaling pathways, involved in the induction of proinflammatory responses. Numerous stimuli including growth factors, cytokines, and mechanical/oxidative stress, contribute to the induction of the activation of Rac1, as well as the creation of the Rac1-GTP protein (an activated form of Rac1). It has been reported that translocation of Rac1-GTP into the membrane-bound cytochrome complex could induce production of both enzymatically active proteins, namely NOX1 and NOX2 (8). These data propose that the activated form of the Rac1 protein could be a vital mediator in the causation of fibrosis and hepatic accumulation of MF-HSC (7).

Statins, as 3-hydroxy-3-methylglutaryl coenzyme A reductase inhibitors, have attracted much attention for their pleiotropic effects. Several lines of evidence indicate that statins exhibit chemopreventive potential against various types of malignancies, including hepatocellular carcinoma. Statins restrain cell growth, reduce proteolysis, and hinder the metastasis process. They also exert potent anti-angiogenic, anti-proliferative, pro-apoptotic, and immunomodulatory potentials. In animal models, statins prevent the activation of hepatic myofibroblasts, induce the apoptosis process, and limit the proliferation of HSCs and production of collagens produced by HSCs (9). Studies have shown that atorvastatin therapy has advantageous consequences on the redox status in rats (10). The attenuation of oxidative stress is one of the proposed pleiotropic effects of statins. Atorvastatin lowers the production of vascular ROS independent of diminishing the cholesterol contents. The impact of atorvastatin on pathology and oxidative stress has been addressed in our previous study (11). Statins not only reduce the generation of ROS but also increase the synthesis of vascular nitric oxide.

Furthermore, some members of the statin family and their metabolites can scavenge the free radical agents within the cells (12). Considering the antioxidant potential of atorvastatin and the impact of the expression of NOX1 and Rac1 on oxidative stress and hepatic fibrogenesis, in this study, we attempted to assess whether the use of atorvastatin affects the expression of NOX1, Rac1-GTP, and Rac1 in BDL-induced cholestasis in rats. For this purpose, we analyzed the markers of oxidative stress and the protein expression of Rac1, Rac1-GTP, and NOX1, which are all involved in the liver fibrogenesis pathways in BDL rats.

## Materials and Methods

***Animal procedure and experimental design***

This study is classified as an experimental interventional study. Adult male Wistar rats (200-250 g) were procured from the Pasteur Institute, Karaj, Iran. The animals were housed in an air-conditioned room with a 12-hr dark/12-hr light cycle at 25°C, while they had free access to standard murine food and tap water. The experimental protocols of the current research were corroborated by the local Ethics Committee of Shahid Sadoughi University of Medical Sciences. A total of 32 rats were randomly allocated into 2 groups: Control groups and BDL groups. Each group was also assigned into two sub-groups for the treatment with either 15 mg/kg/day atorvastatin (Sigma-Aldrich, St. Louis, MO, USA) dissolved in 5% carboxymethyl cellulose (CMC) (2) or the same v/w of 5% CMC was administered to animals through oral gavage from the day post-surgery for 28 days (every day).

The induction of BDL was performed according to the protocols of our previous study (13). Briefly, general anesthesia was carried out by the intraperitoneal injection of ketamine (90 mg/kg) and xylazine (10 mg/kg). Under the sterile condition, the common bile duct was exposed by making an incision in the midline abdominal region of the animals. Afterward, ligation was implemented in two areas using a silk thread and transected between the ligatures (14) Then, cefazolin was employed for the prevention of infection in rats. 

After four weeks, blood specimens were obtained by the heart puncture under deep anesthesia. The collected samples were then centrifuged at 3000 g for 15 min to isolate sera for further experiments. Hepatic tissues were stored at -70˚C to make tissue homogenates for the assessment of oxidative stress parameters and the western blot analysis of NOX1, Rac1-GTP, and Rac1. 

***Evaluation of thiol groups in liver tissue***

 5, 5′ -dithiobis (2-nitrobenzoic acid) or DTNB, also called Ellman’s reagent, is used for the determination of free thiol groups in biological samples. The assay is primarily based on the reaction of the thiol groups with 2-nitrobenzoic acid to produce a disulfide compound and 2-nitro-5-thiobenzoic acid (TNB), which is measured by recording the absorbance of the anion (TNB2-) at 412 nm (15). The analysis of free thiol groups was also examined by the addition of 50 µl Tris, 25 µl DTNB, and 420 µl water (495 µl was applied as a blank) and 5 µl of the sample (final volume = 500 µl). The resultant solution was mildly agitated using a pipette. The samples were poured in the cuvette, placed into a UV-Vis spectrophotometer, and the optical density (OD) of each sample was recorded at the wavelength of 412 nm. The absorbance was measured for each tissue homogenate, and then the values were expressed as nmol/mg protein.

***Measurement of protein carbonylation in liver tissues of rats***

Liver tissue homogenates were treated with 10 mM 2,4 dinitrophenylhydrazine and 2.5 N HCl at room temperature in a dark place for 1 hour. Then, the mixture was treated with 20% trichloroacetic acid and centrifuged. The resultant pellets were rinsed three times with a combination of 99% ethanol and ethyl- acetate at a ratio of 1:1 (v/v). Afterward, the precipitated proteins were dissolved in 6 M guanidine hydrochloride, and their concentration was quantified at the wavelength of 355 nm. The obtained values were expressed as nmol/ mg protein.

***Assessment of catalase activity in liver tissues***

The activity of catalase was evaluated by a technique introduced by Beers *et al*. and Sizer *et al*. (16, 17) in which the decomposition of peroxide is recorded at 240 nm using a spectrophotometer. The sample mixture was prepared in a final volume of 3 ml containing 0.05 M potassium phosphate (pH= 7.4), 0.020 M hydrogen peroxide, and 0.05 ml of liver tissue homogenates (0.05 ml). A reduction in the absorbance of specimens was detected at the wavelength of 240 nm for 3 min. A decline in the rate of the absorbance per min was calculated based on the initial linear portion of the curve. The extinction coefficient of 0.0394 cm-1M-1 was employed to estimate the enzyme activity. The activity of catalase was defined when one unit of the enzyme decomposed one mole of H202 per minute (at 25 ^°^C and pH 7.0), and the final values were presented in IU/mg protein.

***The activity of SOD enzyme in liver tissues***

The measurement of superoxide dismutase (SOD) activity is empirically established on inhibiting the reduction of nitro blue tetrazolium (NBT). In the presence of O_2_ and methionine (as an electron donor), riboflavin is capable of producing superoxide anions, considered the base of SOD assay. The reduction of NBT molecule via superoxide radicals to blue-colored formazan was read at a wavelength of 560 nm, as previously reported in a method by Fridovich and Beauchamp (18).

In this assay, the reaction mixture that contained 1.9 ml phosphate buffer (pH 7.8), 1.17×10^-6^ M riboflavin, 16.8×10^-5^ M NBT, 10-2 M methionine, and tissue homogenates was prepared and proportionally diluted in a total volume of 3 ml. The absorbance of specimens was read at 560 nm for 5 min. The rate of increment in the absorbance per min was calculated based on the initial linear portion of the curve. The extinction coefficient of 0.00436 cm^-1^mol^-1^ was applied for the estimation of the enzyme activity, and the values were expressed as IU/mg protein.

***Western blot analysis***

The protein contents of hepatocytes were extracted by homogenizing liver tissue samples (30 mg) in phosphate-buffered saline [100 mM Tris–HCl, 0.1% SDS, 150 mM NaCl, and 1% NP-40 (pH 7.4) with protease inhibitor cocktail, 1:100; Sigma, St Louis, MO, USA] by incubation of the samples on ice for 30 min and centrifugation at 15000 g (4 ^°^C, 30 min). Protein concentrations were evaluated in the supernatants via the Bradford assay. The extracted proteins (100 µg) were electrophoretically separated on a 12% SDS-polyacrylamide gel and then transferred onto a nitrocellulose membrane. Afterward, the membrane was blocked with 3% non-fat dried milk in Tris-buffered saline, pH 7.4, with 0.05% Tween-20 (TBS/T) for 2 hr and treated with rabbit polyclonal anti-NOX1, mouse monoclonal anti-Rac1-GTP, rabbit primary monoclonal antibody against Rac1, and rabbit polyclonal anti-β actin as an internal control at 4 ^°^C overnight. Concerning the source of primary antibodies utilized in this research, the membrane was treated with goat anti-rabbit/anti-mouse IgG-horseradish peroxidase-conjugated antibodies (1:4000), as secondary antibodies, for 45 min. The size of protein bands predicted for Rac1-GTP (~22 kDa), Rac1 (21 kDa), NOX1 (71 kDa), and β-actin (42 kDa) were examined using a protein marker. The antibody-bound bands were detected by the enhanced chemiluminescence reagent (ECL) using a ChemiDoc system (Syngene GBOX, 680X) and densitometrically semi-quantified by the Quantity GeneTools software (SynGene, V4.1). 

***Statistical analysis***

The obtained experimental values in our experiment were expressed as the means and standard error of the mean (mean±SEM), and differences between groups were calculated by one-way analysis of variance (one way-ANOVA) (19), followed by Tukey’s *post hoc* test for multiple comparisons using the GraphPad Prism software version 5. The *P* value of less than 0.05 was considered statistically significant.

## Results

***Atorvastatin mitigated BDL-induced oxidative stress***

**Figure 1:**
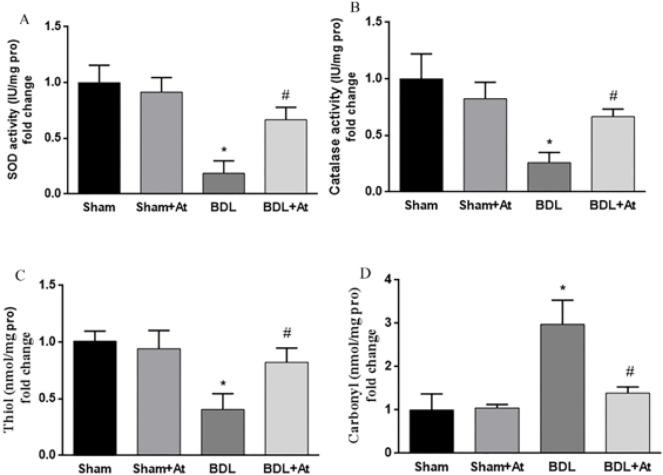
Effect of atorvastatin on the levels of SOD activity (Figure 1A), catalase activity (Figure 1B), thiol group (Figure 1C), and carbonyl group (Figure 1D) in liver tissue of all groups (*P<0.05 vs. the control group; #*P*<0.05 vs. the BDL group). SOD: Superoxide dismutase; At: Atorvastatin; BDL: bile duct ligation

The activity of both SOD and catalase enzymes, as two necessary antioxidant enzymes, along with thiol groups and protein carbonylation, as two biomarkers of protein modifications, that occurred in liver injury, was analyzed. It was observed that the activity of SOD and catalase, as well as the concentration of thiol groups (Figure 1C), was significantly reduced (Figure 1A & 1B), while the protein carbonylation (Figure 1D) was statistically increased in the BDL group when compared to the control group (*P*<0.05). These observations confirmed the increased rate of oxidative stress in hepatic tissue homogenates of the BDL group. Although atorvastatin improved the concentrations of free thiol groups along with the activity of SOD and catalase enzymes in liver tissues (Figure 1A, 1B, & 1C), the carbonyl level of proteins was significantly diminished (Figure 1D).

***The expression of NOX1, Rac1, and Rac1-GTP was declined in the atorvastatin-treated groups***

**Figure 2:**
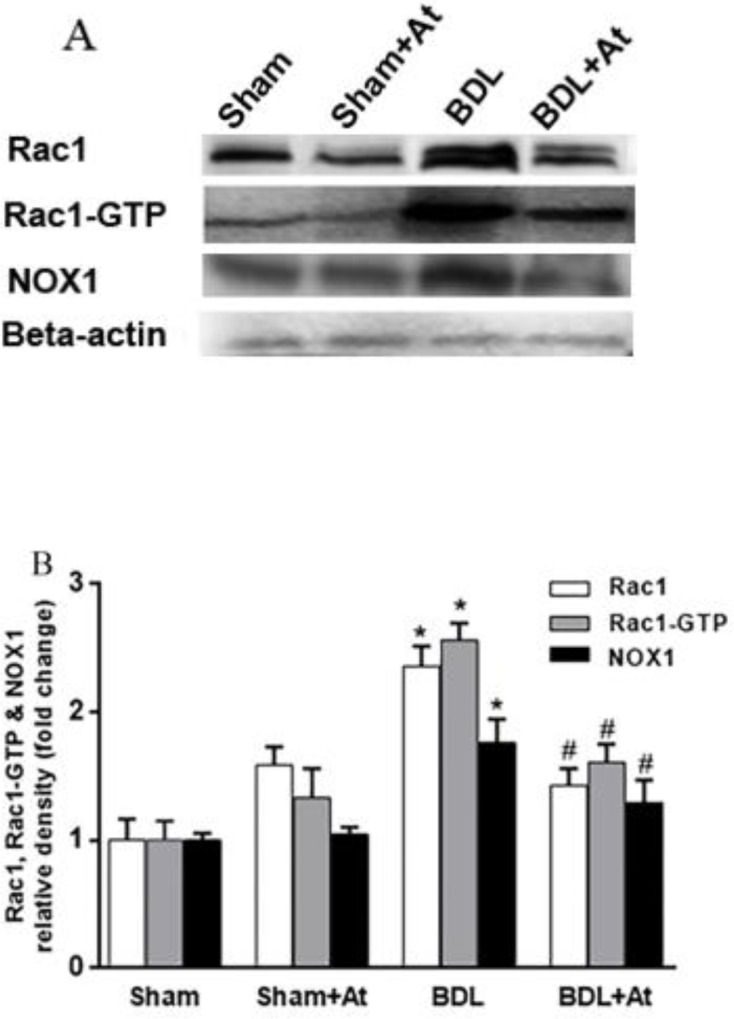
The Expression pattern of NOX1, Rac1-GTP, and Rac1 in the western blot technique (Figure 2A). The relative protein expression of NOX1, Rac1-GTP, and Rac1 (Figure 2B) was evaluated in all experimental groups (*P<0.05 vs. the control group. #*P*<0.05 vs. the BDL group). Rac1: Ras-related C3 botulinum toxin substrate 1; NOX1: NADPH oxidase 1; BDL: bile duct ligation; At: Atorvastatin

The expression rates of NOX1, Rac1-GTP, and Rac1 in liver tissues of all experimental groups were determined by western immunoblot (Figure 2A). The expression levels of NOX1, Rac1-GTP, and Rac1 were significantly up-regulated in liver tissues of the BDL group compared to the control group (*P*<0.05). On the other hand, the expression levels of NOX1, Rac1-GTP, and Rac1 were statistically (*P*<0.05) decreased in liver homogenates of the BDL+At group in comparison with the BDL group (Figure 2B).

## Discussion

Clinical investigations have highlighted the beneficial impacts of atorvastatin therapy on liver fibrosis. However, the mechanism of the beneficial effects of atorvastatin on fibrosis of the liver remained unexplored (2). The present study aimed to unravel the hepatoprotective effect of atorvastatin through the assessment of protein expression of NOX1, Rac1-GTP, and Rac1 in a rat model of BDL. Of note, the severity of oxidative injury to hepatic tissues was evaluated by the measurement of SOD, catalase activity, as well as thiol groups and protein carbonylation as the two markers of protein modifications, which occur in liver injury. Our results showed that atorvastatin treatment notably ameliorated hepatic fibrosis, accompanied by the decreased expression of NOX1, Rac1-GTP, and Rac1 in a rat model of BDL. Atorvastatin also reduced oxidative stress through the modulation of antioxidant enzymes such as SOD and catalase. Atorvastatin regulates the thiol contents and carbonyl groups in the liver of rats with BDL.

BDL has been broadly used as an experimental model for the investigation of biliary cholestasis in rodents, which elevates the systemic oxidative stress. BDL incites HSCs to secrete higher levels of collagen fibrosis, and it causes the accumulation of the extracellular matrix (ECM), which, in turn, leads to liver fibrogenesis and liver cirrhosis (22). 

Our findings have shown that the free thiol groups were significantly decreased in oxidative stress condition, induced by BDL induction. BDL-induced oxidative stress also caused an increase in the generation of the carbonyl groups on protein side chains. In line with our results, Dalle-Donne and colleagues reported elevated levels of carbonyl proteins in multiple human disorders, including fibrosis (23). Our analyses confirmed that the administration of atorvastatin to BDL rats raised the number of free thiol groups, thereby a reduction in disulfide bridges; however; atorvastatin caused an increase in the rate of protein carbonylation significantly. 

Alternatively, we assayed the activities of antioxidant enzymes, including SOD and catalase, as the two leading antioxidant enzymes, serving as endogenous scavengers of free radical in the liver (24, 25). It has been implicated that the activity of SOD and catalase enzymes were significantly lowered in the BDL group when compared to the control group. Notably, the activity of SOD and catalase were elevated dramatically in the BDL+At group, denoting a hepatoprotection role for atorvastatin as it raised the antioxidant capacity.

The effects of atorvastatin on oxidative stress and the activity of enzymes in liver tissue of rats in the control+At group compared to the control group represents a proper dose of atorvastatin (in terms of toxicity) in the development of hepatic fibrosis. 

Hepatic cirrhosis is regarded as the most common cause of death in the world. Patients affected by cirrhosis are more prone to develop hepatic carcinoma compared to the general population. Reversible hepatic wound-healing responses are followed by the development of liver fibrosis and progressive liver dysfunction (26, 27). Generally speaking, hepatic fibrosis is discriminated by unreasonable accumulation and reduced degradation of the ECM. ECM is produced by myofibroblasts, regarded as the primary effector cells in the process of fibrogenesis and liver injury. Accumulation of the ECM proteins and the aggregation of collagen mutually contribute to developing cirrhosis. At the same time, the activation of HSCs is one of the potential sources for these myofibroblasts. HSCs are capable of converting into proliferative and migratory myofibroblasts. At present, practical strategies for hepatic fibrosis include restraining HSC activation and expediting the clearance of activated HSCs (27, 28).

The Rac1 protein controls multiple cellular functions, including the activation of NADPH oxidase, a primary intracellular source for the generation of ROS. It has been identified as the pivotal mediator in the accumulation of MF-HSCs, which are correlated with the progression of hepatic fibrosis (29, 30). Once Rac1 becomes activated, it incites the activation of NOX1 in which the activated NOX1 facilitates the conversion of HSCs into MF cells, subsequently leading to hepatic fibrosis (30). NOX1 is one of the members of the NOX family, which is a vital enzymatic source for ROS production. Numerous homolog-specific mechanisms dominate the activity of this enzyme (31). Activated Rac1 (Rac1-GTP) can directly bind to the NOX1 protein through the TPR site, leading to the regulation of ROS-induced NOX1 activation (30, 32). Recent observations have demonstrated that the activity of the Rac1 protein causes the overproduction of ROS-induced NOX1 activation a then increased HSCs activity, which is thought to play a detrimental role in carbon tetrachloride-induced liver fibrosis (30).

To support this idea, mice deficient for the Rac1 gene showed the suppression of NOX1 and the reduced oxidative stress (33).

Our experiments indicated that the rate of NOX1 expression was significantly elevated in BDL fibrotic rats, and such an increment was in parallel with the overexpression of Rac1 and Rac1-GTP when compared to the control group. However, up-regulation of the proteins mentioned above is declined upon the treatment of BDL fibrotic rats with atorvastatin (BDL+At vs. BDL group).

## Conclusion

This study demonstrated that fibrosis increased the expression of Rac1-GTP, Rac1, and NOX1 in the liver of rats that underwent BDL. Liver fibrosis would gradually result in a reduction in the antioxidant contents (thiols, SOD, and catalase) and an increase in oxidative stress. The treatment with atorvastatin ameliorated liver fibrosis in the BDL+At group by the down-regulation of Rac1-GTP, Rac1, and NOX1, as well as the elevation of antioxidant molecules (thiols, SOD, and catalase). Therefore, our results suggest that atorvastatin is satisfactory for the treatment of liver fibrosis. 
